# Detection of IL28B SNP DNA from Buccal Epithelial Cells, Small Amounts of Serum, and Dried Blood Spots

**DOI:** 10.1371/journal.pone.0033000

**Published:** 2012-03-07

**Authors:** Philippe Halfon, Denis Ouzan, Hacène Khiri, Guillaume Pénaranda, Paul Castellani, Valerie Oulès, Asma Kahloun, Nolwenn Amrani, Lise Fanteria, Agnès Martineau, Lou Naldi, Marc Bourlière

**Affiliations:** 1 Laboratoire Alphabio, Hôpital Ambroise Paré, Marseille, France; 2 Institut Arnault-Tzanck, Saint-Laurent du Var, France; 3 Département d'hépato-gastroentérologie, Hôpital Saint-Joseph, Marseille, France; The Chinese University of Hong Kong, Hong Kong

## Abstract

**Background & Aims:**

Point mutations in the coding region of the interleukin 28 gene (rs12979860) have recently been identified for predicting the outcome of treatment of hepatitis C virus infection. This polymorphism detection was based on whole blood DNA extraction. Alternatively, DNA for genetic diagnosis has been derived from buccal epithelial cells (BEC), dried blood spots (DBS), and genomic DNA from serum. The aim of the study was to investigate the reliability and accuracy of alternative routes of testing for single nucleotide polymorphism allele rs12979860CC.

**Methods:**

Blood, plasma, and sera samples from 200 patients were extracted (400 µL). Buccal smears were tested using an FTA card. To simulate postal delay, we tested the influence of storage at ambient temperature on the different sources of DNA at five time points (baseline, 48 h, 6 days, 9 days, and 12 days)

**Results:**

There was 100% concordance between blood, plasma, sera, and BEC, validating the use of DNA extracted from BEC collected on cytology brushes for genetic testing. Genetic variations in HPTR1 gene were detected using smear technique in blood smear (3620 copies) as well as in buccal smears (5870 copies). These results are similar to those for whole blood diluted at 1/10. A minimum of 0.04 µL, 4 µL, and 40 µL was necessary to obtain exploitable results respectively for whole blood, sera, and plasma. No significant variation between each time point was observed for the different sources of DNA. IL28B SNPs analysis at these different time points showed the same results using the four sources of DNA.

**Conclusion:**

We demonstrated that genomic DNA extraction from buccal cells, small amounts of serum, and dried blood spots is an alternative to DNA extracted from peripheral blood cells and is helpful in retrospective and prospective studies for multiple genetic markers, specifically in hard-to-reach individuals.

## Introduction

Recent studies have demonstrated that host genetics may be useful for predicting the spontaneous clearance of hepatitis C virus during acute hepatitis and the drug response to peginterferon and ribavirin in chronic hepatitis C [1], [2], [3], [4], [5], [6], [7], [8]. The IL28B promoter polymorphism at position -3176 C/T (rs12979860) correlates with a significantly higher rate of spontaneous clearance of the hepatitis C virus (HCV). Individuals with the CC genotype have a two-fold higher sustained virological response (SVR, 55–80%) with peginterferon and ribavirin than those with the CT or TT genotype (SVR, 20–40%) [4], [9].

However, all the IL28B-based studies used whole blood DNA extraction for SNP analysis. DNA circulates freely in blood plasma both in health and in disease, but the source of this DNA remains enigmatic. It is presumed that circulating DNA in healthy subjects is derived from lymphocytes or other nucleated cells [10]. There are several methods for preparing genomic DNA serum, some requiring very small amounts of serum (20–250 µL) [11], [12], [13]. Indeed, DNA for genetic diagnosis has been derived from finger-stick blood samples and genomic DNA from serum roots, cheek scrapings, and urine samples. Oral saline rinses have also been used extensively to collect buccal epithelial cells as a DNA source.

The aim of this study was to investigate the reliability and accuracy of alternative routes of sampling for genetic testing, whether DNA from serum, buccal epithelial cells (BEC), or dried blood spots (DBS) for SNP allele (rs12979860 CC) and to blindly compare these results with those obtained with DNA extracted from whole blood as reference.

## Materials and Methods

Two hundred patients with chronic hepatitis C infection genotype 1 were included from three hepatology units in the south of France (two centres in Marseille and one in Saint Laurent du Var). Mean age was 56±11 years; there were 42 (21%) naïve patients and 40 (20%) non responders. In whole blood, 59 (30%) patients had rs12979860 CC genotype, 107 (53%) had rs12979860 CT genotype, and 34 (17%) had rs12979860 TT genotype.

Written informed consent including for genetic testing was mandatory for inclusion. This study was approved by the French ethics committee “Comité de Protection des Personnes Sud-Méditerranée II” and had the reference number 210R22.

### DBS Preparation

The DBS sample consisted of three drops of 50 µL of whole blood absorbed onto a Protein Saver 903 Card (Whatman, Dassel, Germany) to completely fill 12-mm preprinted circular paper disks and then stored at room temperature. The FTA range of products has been developed to collect, store, and extract nucleic acid from many sample types, including blood, plasma/sera, and buccal smears. After application of the sample to the FTA card, microorganisms are inactivated, cell membranes lysed, and nucleic acids entrapped onto the FTA matrix.

### Genomic DNA extraction

**Figure 1:**
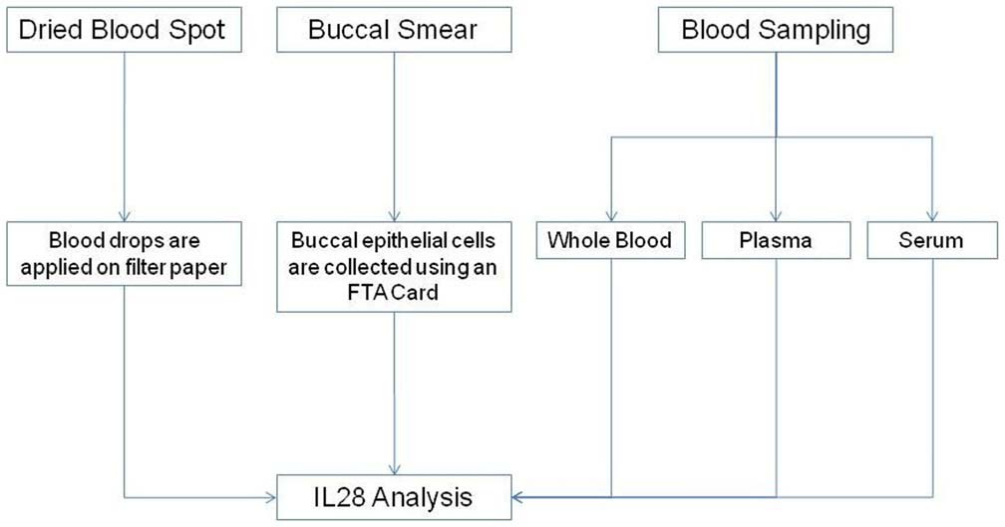
Study Design.

Blood, plasma, and sera samples were extracted with a MagNA Pure device (Roche) according to manufacturer recommendations; 400 µL of each sample type were used. Blood and buccal smears were tested on Whatman system (FTA card and EasyCollect system, respectively). All sampling and tests were performed simultaneously and the amount of DNA was sufficient for molecular analysis. The study design is presented in **Figure 1**.

### EasyCollect system

Cells are captured on the foam applicator by swabbing the oral mucosa and then transferred to indicating FTA Cards [14], [15].

### Sample Processing and DNA Quantification

To test the performance of the different systems, we used CELL Control r-gene (Argene, Verniolle, France) on a Light Cycler 480 II (Roche) according to manufacturer recommendations. The CELL Control r-gene™ is a ready-to-use premix designed to validate results obtained by real time amplification checking the presence of cells on extracted samples. CELL Control r-gene™ determines the number of cells in the sample by quantifying the HPRT1 gene (hypoxanthin phospho-ribosyl transferase) using standards included in the kit. The HPRT1 gene is a housekeeping gene for normalizing gene expression [16].

### Influence of storage at ambient temperature

To simulate postal delay, we assessed the influence of storage at ambient temperature on the different sources of DNA (BEC, DBS, whole blood, and plasma). Ten patients were prospectively sampled and their corresponding sources of DNA were tested after storage at ambient temperature at five time points (baseline, 48 h, 6 days, 9 days, and 12 days). The stability of HPRT1 gene quantification and the IL28B SNPs on BEC, DBS, and plasma was also investigated at these different time points. Whole blood served as a reference source of DNA.

### Testing for IL28B SNPs

The rs12979860 SNPs were analysed in a Fret 5′ allelic discrimination assay using the Roche ‘LightCycler® FastStart DNA Master HybProbe’ with the LightCycler® 1.x/2.0/480 Instruments [9]. A 139-bp-long fragment is amplified with specific primers and analysed in a subsequent melting curve analysis, using a SimpleProbe oligomer specific for the -3176 C allele. The resulting PCR fragments are analysed with a SimpleProbe® in channel 530. The genotypes are identified by running a melting curve with specific melting points (Tm). The wild-type allele T exhibits a Tm of 51.4°C (capillary) or 55°C (plate) while the protective allele variant C shows a significant up shift of 8°C. The melting temperature PCR results are obtained within 50 min (45 cycles and melting curve) with the LightCycler® 1.x/2.0 Instruments and within 80 min (45 cycles and melting curve) with the LightCycler® 480 Instruments.

### Statistical Analysis

Statistical analyses were performed using SAS software (SAS 9.1.3, SAS institute Inc. Cary, NC). Wilcoxon paired test was used to compare results between whole blood, serum, DBS, and BEC. Significance level was set at P<0.05.

## Results

**Figure 2:**
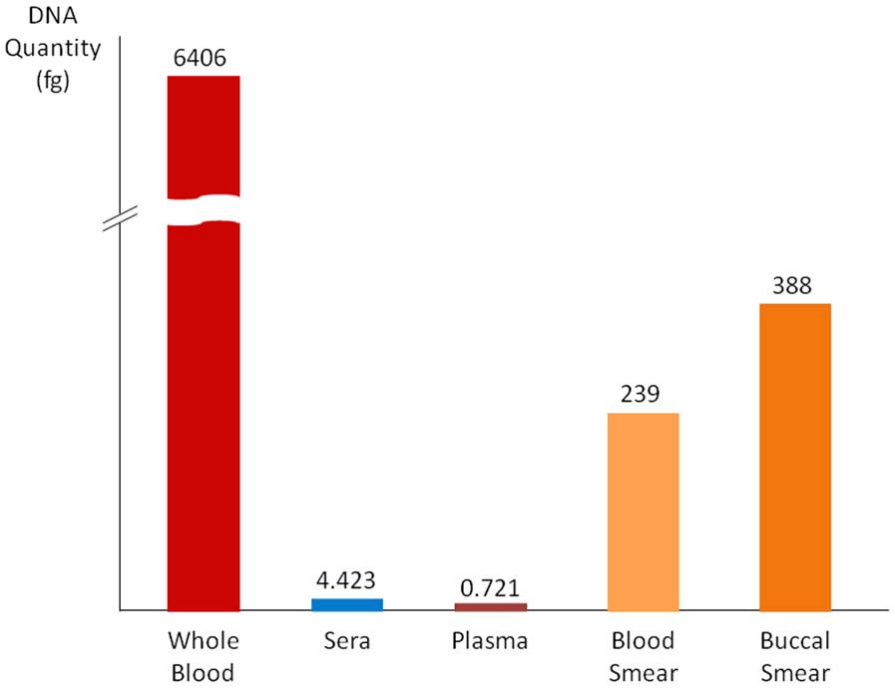
Threshold for DNA detection among different sampling types.

There was 100% concordance in detecting DNA between blood, plasma, sera, and BEC. Regarding the sensitivity of DNA detection of the different samplings, with reference the whole blood, sera and plasma appeared less efficient than DBS and BEC (**Table 1** **and** **Figure 2**).

**Table 1:** Quantity of DNA obtained by different preparation methods and quantification of HPTR-1 gene in blood, sera, and plasma.

Sample	HPRT1 Gene – Median [Range] (Copies)	DNA – Median [Range] (ng)	DNA – Median [Range] (fg)
Blood			
*No dilution*	96900 [95000–98800]	0.006406 [0.006280–0.006532]	6406 [6280–6532]
*Dilution 1/10*	9763 [9100–10426]	0.0006455 [0.0006016–0.0006893]	646 [602–689]
*Dilution 1/100*	686 [590–782]	4.535335E-05 [3.9E-05–5.17E-05]	45.4 [39–51.7]
*Dilution 1/1000*	27.9 [26.2–29.6]	1.84455E-06 [1.73E-06–1.96E-06]	1.84 [1.73–1.96]
*Dilution 1/10000*	1.85 [1.75–1.95]	1.22309E-07 [1.16E-07–1.29E-07]	0.122 [0.116–0.129]
Sera			
*No dilution*	66.9 [52.3–81.5]	4.42296E-06 [3.46E-06–5.39E-06]	4.423 [3.46–5.39)
*Dilution 1/10*	7.63 [5.40–9.86]	5.04442E-07 [3.57E-07–6.51E-07]	0.504 [0.357–0.651]
*Dilution 1/100*	0.228 [0.180–0.276]	1.50738E-08 [1.19E-08–1.82E-08]	0.015 [0.0119–0.0182]
*Dilution 1/1000*	Not detected	Not detected	Not detected
*Dilution 1/10000*	Not detected	Not detected	Not detected
Plasma			
*No dilution*	10.9 [9.9–11.9]	7.20631E-07 [6.54E-07–7.87E-07]	0.721 [0.654–0.787)
*Dilution 1/10*	0.996 [0.852–1.140]	6.58485E-08 [5.63E-08–7.54E-08]	0.0658 [0.0563–0.0754]
*Dilution 1/100*	Not detected	Not detected	Not detected
*Dilution 1/1000*	Not detected	Not detected	Not detected
*Dilution 1/10000*	Not detected	Not detected	Not detected
Blood smear (FTA card)	3620 [3230–4010]	0.0002393 [0.0002135–0.0002651]	239 [214–265]
Buccal smear (EasyCollect)	5870 [5690–6050]	0.0003881 [0.0003762–0.0004000]	388 [376–400]

Blood, sera, and plasma were successively diluted at 10, 100, 1000, and 10000 in PBS. 400 µL of each type of sample were extracted with MagnaPur and eluted in 100 µL of elution buffer. A minimum of 0.04 µL, 4 µL, and 40 µL was necessary to obtain exploitable results respectively for whole blood, sera, and plasma.

Genetic variations in the HPTR1 gene were detected using smear technique in blood smear (3620 copies) as well as in buccal smears (5870 copies). These results are similar to those for whole blood diluted at 1/10.

As 400 µL of each type of sample were extracted with MagnaPur, a minimum of 0.04 µL, 4 µL, and 40 µL was necessary to obtain exploitable results respectively for whole blood, sera, and plasma (**Table 1**).

**Figure 3:**
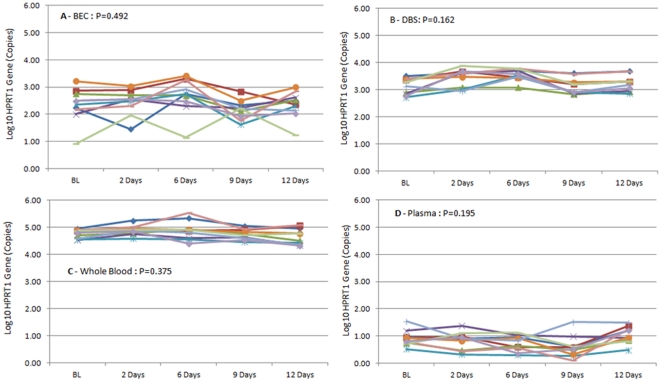
Evolution of HPTR1 gene quantification (Log10 Copies) for buccal smears (3A), DBS (3B), whole blood (3C), and plasma (3D) samples after storage at ambient temperature at five time points (baseline, 48 h, 6 days, 9 days, and 12 days).

**Figure 3** (A,B,C,D) presents the evolution of HPRT1 gene quantification for buccal smears, DBS, whole blood, and plasma samples after storage at ambient temperature at five time points (baseline, 48 h, 6 days, 9 days, and 12 days). No significant variation between each time point (P>0.05, Wilcoxon paired test) was observed for the different sources of DNA.

IL28B SNPs analysis at these different time points showed the same results using the four source of DNA (**Table 2**).

**Table 2:** IL28 SNP according to sampling types (buccal smear, whole blood, plasma, and DBS).

Sample	Buccal Smear	DBS	Whole Blood	Plasma
1	TT	TT	TT	TT
2	TT	TT	TT	TT
3	CC	CC	CC	CC
4	TT	TT	TT	TT
5	CC	CC	CC	CC
6	CC	CC	CC	CC
7	CC	CC	CC	CC
8	CC	CC	CC	CC
9	CC	CC	CC	CC
10	CC	CC	CC	CC

## Discussion

Our study is the first to prospectively compare and validate different DNA sampling sources. Results show that plasma, serum, BEC, or DBS can be used as a source of DNA for IL28B genotyping, with full concordance (100% of sensitivity) with whole blood DNA extraction. The various extraction procedures gave identical SNP genotypes, which confirms that genomic DNA prepared from small amounts of serum or plasma can serve as a template to amplify DNA segments in order to detect genetic alterations, as described by Lin and Floros [11]. Moreover, this study determined the minimal quantity of sera and plasma to be used (4 µL and 40 µL respectively) to reach the cut-off of sensitivity of detection.

It is important to recall that the stability and integrity of genomic DNA depend on storage conditions such as temperature, freeze-thaw cycles, and buffer composition. We observed no influence of storage conditions at ambient temperature, incidently showing postal delay in DNA sample shipping may be avoided. There was no difference in DNA from baseline to 12 days for the different sources of DNA tested (BEC, DBS, whole blood, and plasma). FTA cards, plasma, and sera are appropriate tools for clinical study because of their long term DNA conservation.

The combination of a micro-extraction procedure from serum and PCR genotyping provides a rapid and inexpensive tool for genetic analysis [17]. A small amount of serum (250 µL) can be very informative, since it yields enough DNA to analyse several genetic markers. Note that plasma or serum is more reliable than whole blood for clinical studies, specifically for retrospective studies based on stored DNA and for shipment of sera in clinical trial analysis with centralised laboratories [18]. The present study also evidenced the possibility of using DNA from serum and plasma to simulate serum or plasma utilization for DNA analysis of stored samples.

Cancer patients have a greater amount of circulating DNA than healthy subjects [19]. It seems unlikely that tumoural DNA originates from the lysis of circulating cancer cells because the number of circulating cells usually found in plasma is not high enough to explain the large amount of DNA detected [20]. Apoptosis has been proposed as the origin of circulating DNA, but this mechanism is thought to be lost by proliferating cells [21].

As an alternative to the usual venipuncture technique, several methods have been described that use DBS from finger-puncture or oral swabs collected on DNA-cards [22], [23], [24], [25]. Buccal mucosa cell sampling is almost completely non-invasive and painless, reducing compliance issues. Samples collected generate DNA usable after shipment through conventional mail [26]. Sample collection does not require highly trained personnel or an established ‘cold chain’ to preserve samples until DNA is extracted. FTA™ cards are a viable storage matrix from DNA, and can be extracted to perform Genome Wide Analysis Studies analysis (GWAS) [18]. Moreover, GWAS are commonly used to identify genetic predispositions of many human diseases. Large repositories of biological specimens for clinical and genetic investigations have been established to store material and data for these studies.

Another important issue is that, ideally, tissue sampling should be non-invasive and painless, especially for children and psychiatric groups. Preparation of DNA also needs to be rapid, reliable, and consistent. In any large-scale genetic association project, per-unit cost of DNA preparation becomes high. Also, DNA sample collection is needed in genotyping and in pharmacogenetic studies to understand the inter-individual variability of drug responses.

We also analysed patients' acceptability of the various sampling conditions. Buccal smear had an excellent and acceptability rate, the highest compared with serum and dried blood spot (data not shown).

In conclusion, we report that extracting genomic DNA from small amounts of serum, DBS, and BEC is an appropriate alternative to extracting DNA from peripheral blood cells and offers the possibility to analyse existing specimens. New patterns in genetic markers might be considered using serum as DNA source. DNA cards clearly facilitate genetic and pharmacogenetic testing for routine clinical practice. Moreover, collection and extraction of DNA from buccal smear are a noninvasive, convenient, and cost-effective alternative to blood sampling for participants and researchers, and can be mailed out for self-collection, overcoming geographical impediments. For patients with chronic hepatitis C infection, treatment response may be established through large screening for personalized medicine.
